# Feeding

**Published:** 1887-01-01

**Authors:** 


### Till the Doctor Comes.

[Inquiries and Communications for this column should be addressed, "Physician," care of the Editor of The Hospital.]

### VII.—FEEDING.

Feeding.

Bring the meals on a tray covered with a clean napkin. Prop the patient up with bed-rest or pillow, cover his shoulders, and put a napkin under his chin. If he has to be fed, do not hurry him, wash his mouth afterwards, and make him comfortable. Remove all food from the room at the end of a meal. Do not in severe cases let him sleep too long without food, but in these cases get instructions from the doctor, as sleep may be more beneficial than food. Feeding a patient in an unconscious or semi-conscious state is often a matter of some difficulty : the points to remember are—(1) to give only a small quantity at once ; (2) to pass it well back to the root of the tongue. This often seems a bold plan, but really it is safer than the more timid practice of just passing it between the lips. In the former case it immediately calls into action those muscles concerned in the act of swallowing, and usually at once disappears ; in the latter it remains gurgling and accumulating in the mouth, until it is perhaps suddenly drawn in the wind-pipe by the patient taking a deep breath, when it gives rise to alarming symptoms of choking. Sometimes it is possible, if the patient keeps his teeth firmly clenched, to pass one finger between the teeth and cheek, and draw the cheek outward, thus forming a pouch into which the nourishment may be poured ; then by withdrawing the finger and keeping the head low, the liquid may often be pressed into the centre and back part of the mouth, when it is immediately swallowed. In these cases only liquid nourishment must be given. In any severe case it is advisable to get the medical man in charge of the patient to order in writing the diet of the patient, and this must be strictly adhered to. Soda-water and milk (3 parts to 1) is a most refreshing drink for a feverish patient. In cases of serious or exhausting disease, always give the patient a little warm food—warm milk or arrowroot—with perhaps a little brandy in it just before he settles down for the night. Also have ready for him, when he wakes early in the morning, a little warm nourishment, and keep rather more fire going, as it is at this time that the period of lowest temperature of the body is reached, and a little extra warmth should be furnished, that he may quickly rally from any approaching exhaustion.

Preservation of Ice.

Ice in many cases of illness is so valuable that any hint as to its preservation is useful. To prevent it from melting too quickly the great need is to drain its water away as quickly as it forms. This is best done by getting a piece of coarse flannel with large, open meshes, and tying it round the mouth of an ordinary tumbler, so as to leave a cup-shaped depression of flannel to half the depth of the tumbler. This flannel cup may be filled with small pieces of ice, and another piece of flannel put over the top. A reserve supply in a cool place outside the bedroom may be secured by making a flannel cup on the above plan, in a jug, and filling it with ice, care being taken that there is space enough below the bag to allow the water to collect, and leave the ice dry. Ice is best broken into small pieces by the point of a strong needle or skewer.

(*To be continued.*)

